# Biofilm Detection in Bacterial Isolates From Musculoskeletal Infections: A Comparative Study of Tissue Culture Plate and Congo Red Agar Methods With Antimicrobial Susceptibility Correlation

**DOI:** 10.7759/cureus.106879

**Published:** 2026-04-12

**Authors:** Priyank Trivedi, Shreeraj Talwadekar, Gita Nataraj

**Affiliations:** 1 Department of Microbiology, Seth Gordhandas Sunderdas Medical College and King Edward Memorial Hospital, Mumbai, IND

**Keywords:** antimicrobial resistance, biofilm formation, congo red agar, musculoskeletal infections, tissue culture plate method

## Abstract

Background

Musculoskeletal infections are a significant cause of morbidity and are frequently associated with prolonged hospital stay, increased healthcare costs, and poor functional outcomes. A major contributing factor to the persistence and recurrence of these infections is the ability of microorganisms to form biofilms. Biofilm-associated bacteria exhibit enhanced resistance to host immune responses and antimicrobial agents, making their detection clinically important. This study aimed to evaluate the prevalence of biofilm formation in bacterial isolates from musculoskeletal infections, compare phenotypic detection methods, and assess their antimicrobial susceptibility patterns.

Methods

This prospective study was conducted over a period of 12 months in the Bacteriology division of a tertiary care hospital. A total of 105 non-duplicate bacterial isolates obtained from 90 cases of musculoskeletal infections were included. Biofilm detection was performed using two phenotypic methods: Congo red agar (CRA) and the tissue culture plate (TCP) method, a standardized microtiter plate assay. The TCP method was considered the reference standard. Antimicrobial susceptibility testing was carried out using the Kirby-Bauer disc diffusion method in accordance with Clinical and Laboratory Standards Institute (CLSI) guidelines.

Results

The most common clinical condition was infected/open fractures (18; 20.0%), followed by trauma (7; 7.8%), osteomyelitis, implant infections, and joint infections (6; 6.7% each), while miscellaneous cases accounted for 37 (41.1%). *Staphylococcus aureus *was the most frequently isolated organism (26; 24.8%), followed by *Klebsiella pneumoniae* (22; 21.0%) and *Pseudomonas aeruginosa* (15; 14.3%). Biofilm formation was detected in 16 (15.2%) isolates by TCP method, compared to seven (6.7%) by CRA method, with CRA missing nine biofilm-producing isolates. Among organisms, biofilm production was highest in* Enterobacter *spp. and *Proteus *spp. (33.3% each), followed by *Acinetobacter baumannii (*28.6%), coagulase-negative staphylococci(25.0%), and *S. aureus* (19.2%). Biofilm-producing isolates were more commonly observed with joint infections (50.0%) and spine infections (40.0%). Among Gram-negative isolates, maximum sensitivity was observed to carbapenems (40; 64.0%) and gentamicin (34; 55.0%), whereas resistance was high for cephalosporins and fluoroquinolones. Among Gram-positive isolates, all were sensitive to linezolid and teicoplanin (100%), followed by gentamicin (78.0%). Biofilm-producing isolates demonstrated comparatively higher resistance to commonly used antibiotics than non-biofilm producers; however, this difference was not statistically significant.

Conclusion

Biofilm formation is an important virulence factor in musculoskeletal infections and contributes to antimicrobial resistance and chronicity. The TCP method is a more sensitive and reliable technique compared to CRA for the detection of biofilm production. Routine screening for biofilm formation, along with appropriate antimicrobial stewardship, can aid in better clinical management and improved patient outcomes.

## Introduction

Musculoskeletal infections are a common clinical problem associated with significant morbidity, as well as considerable financial and psychosocial burden on patients and healthcare systems [1]. These infections include a wide spectrum of conditions such as osteomyelitis, septic arthritis, implant-associated infections, and post-traumatic infections, necessitating a thorough understanding of their pathogenesis and management. Of particular concern is the role of bacterial biofilm formation, as biofilm-mediated infections are often difficult to diagnose and are associated with poor therapeutic outcomes [2].

A variety of microorganisms are implicated in musculoskeletal infections, including coagulase-negative staphylococci (CONS)*, Staphylococcus aureus, Streptococcus *spp., *Enterococcus *spp., Gram-negative bacilli, and anaerobes [3]. Among these, staphylococcal species, especially *S. aureus *and *S. epidermidis*, are the most commonly identified biofilm-forming organisms. It is estimated that more than 99% of bacteria exist in biofilms, particularly in clinical settings involving indwelling medical devices [4]. Furthermore, *S. epidermidis, S. aureus, *and* Pseudomonas aeruginosa* together account for nearly 75% of biofilms associated with medical devices [5]. In prosthetic joint infections, biofilm-forming bacteria adhere to implant surfaces, bone, and surrounding tissues, and may also be present as aggregates within joint fluid [6].

Biofilm is defined as a structured community of microbial cells enclosed in a self-produced extracellular polymeric matrix that adheres to inert or living surfaces. This matrix facilitates bacterial survival by limiting antibiotic penetration and protecting organisms from host immune responses. The resistance exhibited by biofilm-associated organisms may be intrinsic, due to altered metabolic states, or acquired through horizontal gene transfer within the biofilm environment [7]. The extracellular polymeric substance (EPS) matrix further contributes to antimicrobial resistance by impeding drug diffusion and promoting the persistence of virulent strains. For instance, genes such as ndvB have been implicated in biofilm-associated resistance in *P. aeruginosa* [8].

Biofilm formation is a complex and regulated multi-step process involving initial adsorption of molecules to surfaces, bacterial adhesion, production of EPS, microcolony formation, and eventual maturation of the biofilm [9]. In musculoskeletal infections, particularly those involving prosthetic devices, biofilms play a critical role in persistent infection, delayed wound healing, and treatment failure. However, the exact interplay between biofilms, host factors, and environmental influences requires further clinical and scientific elucidation [10].

Accurate detection of biofilm formation is essential for effective management of such infections. Various methods have been developed for biofilm detection and quantification, including tube methods, Congo red agar (CRA), microtiter plate assays, radiolabeling, and microscopy. Among these, the microtiter (tissue culture) plate method is widely regarded as a reliable and reproducible technique for biofilm assessment, with both terms often used interchangeably in the literature. However, no standardized protocol currently exists, and results may vary depending on culture conditions and environmental factors. Additionally, both phenotypic and genotypic methods are important for comprehensive evaluation, as biofilm expression can be influenced by factors such as nutrient composition and environmental stress.

In view of the clinical importance of biofilm-associated infections and the limitations of existing diagnostic methods, the present study was undertaken to evaluate biofilm formation in bacterial isolates recovered from musculoskeletal infections using phenotypic techniques. The present study aims to (a) evaluate the prevalence of biofilm formation in bacterial isolates from musculoskeletal infections, (b) compare CRA and tissue culture plate (TCP) methods for biofilm detection, with TCP considered as the reference standard, and (c) correlate biofilm formation with antimicrobial susceptibility patterns.

## Materials and methods

Study design

This prospective study was conducted in the Bacteriology Division of the Department of Microbiology at Seth Gordhandas Sunderdas Medical College and King Edward Memorial Hospital, Mumbai, India, over a period of 12 months (January 2020 to December 2020) following institutional ethics approval (EC/91/2019). The sample size was calculated using Cochran’s formula, considering a prevalence of biofilm-producing organisms of 80% [9], a precision of 8%, and a population size of approximately 1000 musculoskeletal infection cases per year. The calculated sample size was 246 for an infinite population, which was adjusted to 105 isolates for the present study. Finally, 105 non-duplicate bacterial isolates obtained from 90 cases of musculoskeletal infections were included. The difference between cases and isolates was due to polymicrobial infections, as 13 cases yielded two organisms and one case yielded three organisms. Non-duplicate bacterial isolates obtained from clinically diagnosed cases of musculoskeletal infections were included in the study. Isolates from patients who had received antibiotic therapy within the preceding seven days were excluded from the study.

Biofilm detection methods

Biofilm formation was assessed using two phenotypic methods: the CRA method [11] and the TCP method [12].

The CRA method was performed as described by Freeman et al. [11]. The medium consisted of brain heart infusion broth supplemented with sucrose, agar, and Congo red dye. Plates were inoculated and incubated aerobically at 37°C for 24 hours. Black colonies with a dry crystalline appearance were considered positive for biofilm production, whereas pink colonies or black colonies lacking dry consistency were considered negative.

The TCP method used in this study is a standardized form of the microtiter plate assay. Both utilize 96-well polystyrene microtiter plates, and there is no structural difference between a “tissue culture plate” and a “microtiter plate.” The distinction lies in the standardized protocol and interpretation criteria described by Stepanović et al., which were followed in the present study to ensure reproducibility [12]. A 0.5 McFarland suspension of each isolate was prepared in trypticase soy broth and incubated for 24 hours. Aliquots were inoculated into sterile 96-well polystyrene plates along with appropriate positive and negative controls. After incubation, wells were washed with phosphate-buffered saline, heat-fixed, and stained with 0.1% crystal violet. The stain was eluted using 33% acetic acid, and optical density was measured at 570 nm using an enzyme-linked immunosorbent assay (ELISA) reader. All samples were processed in triplicate. Biofilm production was interpreted based on optical density cut-off (ODc), calculated as the mean optical density of the negative control plus three standard deviations (SDs). Values above ODc were considered positive for biofilm formation.

Antimicrobial susceptibility testing

Antimicrobial susceptibility testing was performed using the Kirby-Bauer disc diffusion method on Mueller-Hinton agar, following the Clinical and Laboratory Standards Institute (CLSI) 2021 guidelines [13]. A panel of antibiotics was tested for both Gram-positive and Gram-negative organisms. Quality control was ensured using standard American Type Culture Collection (ATCC) strains, including *S. aureus* ATCC 25923, *P. aeruginosa* ATCC 27853, *Escherichia coli* ATCC 25922, and *Enterococcus faecalis* ATCC 29212.

Statistical analysis

Data were entered into Microsoft Excel (Microsoft Corp., Redmond, WA, USA) and analyzed using IBM SPSS Statistics for Windows, Version 27 (Released 2019; IBM Corp., Armonk, New York, United States). Continuous variables are presented as mean ± SD, while categorical variables are expressed as frequencies and percentages. Fisher’s exact test was used to compare qualitative variables. A p-value <0.05 was considered statistically significant.

## Results

The majority of cases were infected/open fractures (18; 20.0%). Trauma accounted for seven (7.8%) cases, while osteomyelitis, implant infections, and joint infections each constituted six (6.7%) cases. Spine infections were observed in five (5.6%) cases, whereas sinus (3; 3.3%) and suture site infections (2; 2.2%) were less common. Miscellaneous cases accounted for 37 (41.1%) of the total (Table 1).

**Table 1:** Clinical Spectrum of Cases (n = 90).

Diagnosis	n (%)
Fractures (infected/open)	18 (20.0)
Osteomyelitis	6 (6.7)
Trauma	7 (7.8)
Spine infections	5 (5.6)
Implant infections	6 (6.7)
Joint infections	6 (6.7)
Sinus	3 (3.3)
Suture site infection	2 (2.2)
Miscellaneous	37 (41.1)
Total	90 (100)

*S. aureus* was the most common isolate (26; 24.8%), followed by *Klebsiella pneumoniae* (22; 21.0%) and *P. aeruginosa *(15; 14.3%). CONS accounted for 12 (11.4%) isolates. *Acinetobacter baumannii* and *E. coli *each contributed seven (6.7%) isolates. *Enterobacter *spp., *Proteus *spp., *Providentia *spp., and *Enterococcus *spp.were isolated in three (2.9%) cases each, while *Streptococcus *spp. accounted for two (1.9%) isolates. Other non-fermenting Gram-negative bacilli and *Citrobacter *spp. were least common, with one (1.0%) isolate each (Table 2).

**Table 2:** Distribution of Bacterial Isolates (n = 105). CONS: coagulase-negative staphylococci; GNB: Gram-negative bacilli

Organism	n (%)
Staphylococcus aureus	26 (24.8)
Klebsiella pneumoniae	22 (21.0)
Pseudomonas aeruginosa	15 (14.3)
CONS	12 (11.4)
Acinetobacter baumannii	7 (6.7)
Escherichia coli	7 (6.7)
*Enterobacter *spp.	3 (2.9)
*Proteus* spp.	3 (2.9)
*Providentia *spp.	3 (2.9)
*Enterococcus* spp.	3 (2.9)
*Streptococcus *spp.	2 (1.9)
Other non-fermenting GNB	1 (1.0)
*Citrobacter* spp.	1 (1.0)
Total	105 (100)

Biofilm formation was detected in 16 (15.2%) isolates by the TCP method, whereas the CRA method identified seven (6.7%) isolates as biofilm producers (Table 3).

**Table 3:** Biofilm Detection by Different Methods (n = 105).

Method	n (%)
Tissue Culture Plate (TCP)	16 (15.2)
Congo Red Agar (CRA)	7 (6.7)

Biofilm production was highest in *Enterobacter *spp. and* Proteus* spp. (one each; 33.3%), followed by *A. baumannii *(2; 28.6%) and CONS (3; 25.0%). *S. aureus* accounted for five (19.2%) biofilm-producing isolates, while *P. aeruginosa *contributed two (13.3%) and *K. pneumoniae* two (9.1%). No biofilm production was observed in *E. coli,* *Enterococcus *spp., or other isolates (Table 4).

**Table 4:** Biofilm Production Among Isolates (n = 105). CONS: coagulase-negative staphylococci

Organism	Biofilm Positive, n (%)
Acinetobacter baumannii	2 (28.6)
Escherichia coli	0 (0)
*Enterobacter* spp.	1 (33.3)
*Enterococcus *spp.	0 (0)
Klebsiella pneumoniae	2 (9.1)
CONS	3 (25.0)
Staphylococcus aureus	5 (19.2)
Pseudomonas aeruginosa	2 (13.3)
*Proteus *spp.	1 (33.3)
Others	0 (0)
Total	16 (15.2)

Biofilm-producing isolates were most frequently observed in joint infections and suture site infections (3; 50.0% and 1; 50.0%, respectively), followed by spine infections (2; 40.0%). Osteomyelitis and implant infections each showed one (16.7%) case, while fractures accounted for two (11.1%) cases. Miscellaneous cases contributed six (16.2%). No biofilm production was observed in trauma and sinus-related cases (Table 5).

**Table 5:** Distribution of Biofilm Producers Across Clinical Categories (n = 90).

Clinical Category	Biofilm Positive, n (%)
Fractures	2 (11.1)
Osteomyelitis	1 (16.7)
Trauma	0 (0)
Spine	2 (40.0)
Implant infection	1 (16.7)
Joint infection	3 (50.0)
Sinus	0 (0)
Suture site infection	1 (50.0)
Miscellaneous	6 (16.2)
Total	16 (17.8)

Among Gram-negative organisms (n = 62), maximum sensitivity was observed to carbapenems (40; 64.0%), followed by gentamicin (34; 55.0%). Sensitivity to fluoroquinolones ranged from 11 to 22 (18-36%), while third- and fourth-generation cephalosporins showed minimal sensitivity (1-2; 2-3%). Among Gram-positive organisms (n = 43), all isolates were sensitive to linezolid and teicoplanin (43; 100% each). Gentamicin showed sensitivity in 34 (78.0%) isolates, followed by cotrimoxazole (25; 58.0%) and clindamycin (18; 42.0%). Lower sensitivity was observed for ciprofloxacin and erythromycin (8; 19.0% each), while no isolates were sensitive to tetracycline (0%) (Table 6).

**Table 6:** Summary of Antimicrobial Sensitivity. Cephalosporins (third/fourth generation) include ceftriaxone, ceftazidime, and cefepime. Fluoroquinolones include ciprofloxacin and levofloxacin. Values are presented as ranges, indicating variability in sensitivity across antibiotics within each class. * Variable across ciprofloxacin and levofloxacin; ^#^ Very low sensitivity across ceftriaxone, ceftazidime, and cefepime.

Organisms	Antibiotic Class	Sensitivity, n (%)
Gram-Negative Organisms (n = 62)	Carbapenems	40 (64.0)
	Gentamicin	34 (55.0)
	Fluoroquinolones	11-22 (18-36)*
	Cephalosporins (third/fourth)	1-2 (2-3)^#^
Gram-Positive Organisms (n = 43)	Linezolid	43 (100)
	Teicoplanin	43 (100)
	Gentamicin	34 (78.0)
	Cotrimoxazole	25 (58.0)
	Clindamycin	18 (42.0)
	Ciprofloxacin	8 (19.0)
	Erythromycin	8 (19.0)
	Tetracycline	0 (0)

Biofilm-producing isolates demonstrated a trend toward higher resistance compared to non-biofilm producers across most antibiotic classes; however, these differences were not statistically significant. Sensitivity to carbapenems was higher among biofilm producers (75.0%) compared to non-biofilm producers (62.9%), but the difference was not significant (p = 0.408). Aminoglycoside sensitivity was comparable between the two groups (62.5% vs. 65.2%; p = 0.999). Similarly, lower sensitivity to fluoroquinolones was observed in biofilm producers (25.0%) compared to non-biofilm producers (32.6%), though this difference was not statistically significant (p = 0.770). Cephalosporins (third/fourth generation) showed low sensitivity in both groups (12.5% vs. 9.0%; p = 0.647). Overall, although biofilm-producing isolates tended to exhibit reduced antimicrobial susceptibility, no statistically significant association was found between biofilm formation and antibiotic resistance in this study (Table 7).

**Table 7:** Comparison of Antimicrobial Susceptibility Between Biofilm-Producing and Non-biofilm-Producing Isolates.

Antibiotic Class	Biofilm Producers (n = 16) Sensitive, n (%)	Non-biofilm Producers (n = 89) Sensitive, n (%)	Fisher’s Exact Test (P-value)
Carbapenems	12 (75.0)	56 (62.9)	0.408
Aminoglycosides	10 (62.5)	58 (65.2)	0.999
Fluoroquinolones	4 (25.0)	29 (32.6)	0.770
Cephalosporins (third/fourth)	2 (12.5)	8 (9.0)	0.647

## Discussion

Musculoskeletal infections continue to pose a significant therapeutic challenge, particularly due to the involvement of biofilm-forming organisms, which contribute to persistent infection, implant failure, and increased antimicrobial resistance. In the present study, 105 isolates from 90 cases were analyzed to evaluate the clinical spectrum, microbiological profile, biofilm formation, and antimicrobial susceptibility patterns.

With respect to clinical distribution (Table 1), infected/open fractures were the most common defined clinical entity (20.0%), followed by trauma, osteomyelitis, implant infections, and joint infections. A large proportion of cases fell under miscellaneous (41.1%), reflecting the diverse presentation of musculoskeletal infections. Similar observations have been reported in orthopedic infection studies, where trauma and fracture-related infections constitute a significant burden due to increased exposure and surgical interventions [14].

The microbiological profile (Table 2) revealed *S. aureus *(24.8%) as the predominant organism, followed by *K. pneumoniae *(21.0%) and *P. aeruginosa* (14.3%). These findings are consistent with studies by Arunshankar et al., where *S. aureus* (20%) was the most common pathogen, followed by CONS and *P. aeruginosa* [15]. Similarly, Gupta et al. reported *S. aureus* as the leading isolate, followed by Gram-negative organisms such as *K. pneumoniae* and *A. baumannii* [14]. A review by Peel et al. also highlighted the predominance of *S. aureus* and CONS in prosthetic joint infections [16].

Biofilm formation (Table 3) was detected in 15.2% of isolates by the TCP method, compared to 6.7% by CRA. The superior sensitivity of TCP over CRA observed in this study is in agreement with previous studies by Mathur et al. and Stepanović et al., which have established TCP as the gold standard phenotypic method for biofilm detection [12,17]. The lower sensitivity of CRA may be attributed to its subjective interpretation and variability across different bacterial species, as also reported by Thakkar et al. [18] and Shrestha et al. [19].

Among individual organisms (Table 4), biofilm production was highest in* Enterobacter* spp. and *Proteus *spp. (33.3%), followed by *A. baumannii *(28.6%), CONS (25.0%), and *S. aureus* (19.2%). These findings are comparable to studies by Singhai et al. and Folliero et al., which identified *Staphylococcus, Pseudomonas, Klebsiella, *and *Acinetobacter *as common biofilm producers in device-related infections [20,21]. However, the overall prevalence of biofilm producers in the present study (15.2%) was lower than that reported in literature, possibly due to the inclusion of intraoperative samples rather than exclusively device-associated infections [9,22].

The distribution of biofilm producers across clinical categories (Table 5) showed the highest prevalence in joint infections (50.0%) and spine infections (40.0%), followed by osteomyelitis and implant infections (16.7% each). This is consistent with the known propensity of biofilms to form on prosthetic surfaces and poorly vascularized tissues, as highlighted by Zimmerli and Moser [23]. The absence of biofilm production in trauma and sinus cases may be explained by the acute nature of these conditions, where biofilm maturation has not yet occurred.

Antimicrobial susceptibility patterns (Table 6) demonstrated that Gram-negative organisms were most sensitive to carbapenems (64.0%) and aminoglycosides, while showing high resistance to cephalosporins and fluoroquinolones. Similar findings have been reported by Cepas et al. and Singhai et al., where carbapenems and piperacillin-tazobactam were the most effective agents against Gram-negative biofilm-associated infections [21,24]. Among Gram-positive organisms, 100% sensitivity to linezolid and teicoplanin was observed, consistent with studies by Arunshankar et al. and Brady et al., which emphasize the efficacy of glycopeptides and oxazolidinones in treating such infections [15,25]. The high resistance to fluoroquinolones and macrolides observed in the present study reflects the growing concern of antimicrobial resistance.

Comparison between biofilm and non-biofilm producers (Table 7) showed higher resistance among biofilm-forming isolates, although the difference was not statistically significant. This trend is well documented, as biofilms confer protection through EPS, reduced metabolic activity, and enhanced horizontal gene transfer, leading to multidrug resistance [9].

Overall, the findings of the present study are in concordance with existing literature and reinforce the clinical significance of biofilm detection in musculoskeletal infections. However, certain limitations should be acknowledged. The relatively small sample size and single-center design may limit the generalizability of the findings. Additionally, molecular characterization of biofilm-associated genes was not performed, which could have provided deeper insight into the mechanisms of biofilm formation. Patient demographic details and clinical outcomes were not analyzed, as the study was isolate-based and included polymicrobial infections, limiting direct correlation with clinical variables. These factors should be considered while interpreting the results. Early identification of biofilm-forming organisms, along with appropriate antimicrobial therapy, remains essential for improving patient outcomes and reducing the burden of chronic and recurrent infections.

## Conclusions

Biofilm formation plays a significant role in musculoskeletal infections, contributing to persistence and antimicrobial resistance. *S. aureus* was the predominant isolate, with Gram-negative organisms also frequently implicated. The TCP method demonstrated higher sensitivity than CRA for detecting biofilm production. Higher biofilm prevalence was observed in joint and spine infections. Gram-negative isolates showed maximum sensitivity to carbapenems and aminoglycosides, while Gram-positive organisms were uniformly sensitive to linezolid and teicoplanin. Biofilm producers demonstrated increased resistance, though not statistically significant. Routine detection of biofilm may improve targeted therapy and clinical outcomes in musculoskeletal infections.
